# Selected Papers from the First International Symposium on Future ICT (Future-ICT 2019) in Conjunction with the 4th International Symposium on Mobile Internet Security (MobiSec 2019)

**DOI:** 10.3390/s21010265

**Published:** 2021-01-03

**Authors:** Giovanni Pau, Hsing-Chung Chen, Fang-Yie Leu, Ilsun You

**Affiliations:** 1 Faculty of Engineering and Architecture, Kore University of Enna, 94100 Enna, Italy; giovanni.pau@unikore.it; 2 Department of Computer Science and Information Engineering, Asia University, Taichung 41354, Taiwan; cdma2000@asia.edu.tw; 3 Department of Computer Science, Tunghai University, Taichung 40799, Taiwan; leufy@thu.edu.tw; 4 Department of Information Security Engineering, Soonchunhyang University, 22 Soonchunhyang-ro, Shinchang-myeon, Asan-si 31538, Choongchungnam-do, Korea

The International Symposium on the Future ICT (Future-ICT 2019) in conjunction with the 4th International Symposium on Mobile Internet Security (MobiSec 2019) has been held on 17–19 October 2019 in Taichung, Taiwan. The symposium provided academic and industry professionals an opportunity to discuss the latest issues and progress in advancing smart applications based on future ICT and its relative security. The symposium aimed to publish high-quality papers strictly related to the various theories and practical applications concerning advanced smart applications, future ICT, and related communications and networks. Furthermore, it was expected that the symposium and its publications could be a trigger for further related research and technology improvements in this subject matter.

The authors of conference papers falling in the Sensors’ scope at this symposium have been invited to submit the extended versions to this Special Issue for publication. Moreover, new papers strictly related to the conference themes have also been welcome. Among the 18 submissions, the guest editors picked 12 high-level contributions for publication after several rounds of review carried out by invited experts.

The authors of [1] introduce a new algorithm and its systolic composition for digital normalized cross-correlation, based on the statistical characteristic of an inner-product. A relationship between the inner-product in cross-correlation and a first-order moment is acquainted. Subsequently, digital normalized cross-correlation is molded in a novel estimation method that essentially comprises the first-order moment. As the first-order moment’s algorithm can be realized by systolic structure, the authors design a systolic array for normalized cross-correlation with a seldom multiplier for its fast hardware implementation. The comparison with other approaches highlights the encouraging performance of the proposed method.

A direct byte read (DRB) durable hybrid RAM disk (DHRD) scheme for hybrid storage systems, composed of RAM disk and SSD, is presented in [2]. The proposed solution implements a byte-range interface, agreeable with present interfaces, and can be practiced with buffered writes. Experimental evaluations are conducted utilizing various benchmarks that apply to various systems delivering dense I/O operations. For the hybrid storage device, the proposed scheme plays three to five times quicker throughputs than other approaches and can also diminish the accomplishment times of multimedia files read and write processing.

The authors of [3] advance a simplistic option for determining the fine aerosols with a diameter of less than 2.5 microns ($PM_{2.5}$) concentration, in which a set of image processing schemes and simple linear regression are applied. The suggested approach practices images with a high and low $PM_{2.5}$ concentration to differentiate these images. Examinations are carried out to validate the proposed approach employing an image data set and an open $PM_{2.5}$ concentration data set. The results show that the proposed approach delivers the best performance compared to other solutions.

A census transform with the Haar wavelet (CTHW) method, enhancing the efficiency with a wavelet transform, and an adaptive window census transform (AWCT), to allow the conversion window size adjusted for every point, are introduced in [4]. The suggested CTHW can produce a more favorable result with a small window size and be pleasantly employed to a low computational resource system. Besides, AWCT delivers better performance in lessening the running times with satisfactory quality.

The authors of [5] propose an effective multidimensional secret data-embedding scheme based on the mini SuDoKu matrix. The reference matrix is extended to multi-dimension to achieve even higher embedding capacity while still maintaining adequate security and efficiency. The proposed scheme is compared with other solutions, and the empirical results show that it achieves higher dB in terms of image quality and two bits per pixel in terms of the embedding capacity. Moreover, the proposed algorithm’s time consumption is smaller than half of the conventional approach.

The authors of [6] propose the improvement for Error-correction codes (ECC) capability inside the page and RAID parity management outside the page, counting on lossless data compression. The adaptive ECC method can lessen the size of a source length in relationship to the compression ratio. The experimental results prove that adaptive ECC can improve error recovery’s efficacy, thus achieving high reliability.

A secret image sharing solution based on a new maze matrix is presented in [7]. A pair of different cover images are utilized to carry secret data, and a pair of shadow images are formed following the supervision of the maze matrix. The secret data is obtained if both true shadows are manifested. Performance evaluations show that the detection ratio is 43% for cases in which a single shadow is tampered with, while it is 72% for cases in which both shadows tamper.

The authors of [8] propose a multi-labeled hierarchical classification (MLHC) learning model with hierarchical groups that attack employing a machine-learning algorithm to identify freak operations of the in-vehicle network. The suggested method can execute prompt decisions about an attack or favorable circumstances for in-vehicle networks by learning the CAN traffic, and it can record further accurate knowledge when an attack is recognized. The simulation outcomes reveal that the proposed approach delivers excellent efficiency.

A second-order Markov model for predicting the frame-level communication failures in an Industrial Wireless Sensor Network (IWSN), based on the preliminary tracks collected in a real-world factory, is introduced in [9]. The proposed model affords a more detailed explanation of the communication quality in IWSNs than traditional approaches. The simulation results prove that the suggested method increases the expected communication reliability compared to that obtained employing the original independent error model.

The authors of [10] suggest a new signature method employing certificateless public key cryptography (CL-PKC) to produce and validate a message’s signature in an IoT environment. The advanced system is a certificateless aggregate arbitrated signature, and the gateway aggregates the signatures of messages created by the device group to lessen the volume of the whole signature. Validations show that the authors’ solution can resolve the difficulties caused by public key replacement attacks and malicious key generation center (KGC), adding arbitrated signatures of the gateway to increase non-repudiation.

A global navigation satellite system (GNSS)-based crowd-sensing policy for distinct geographic regions, useful to determine how many targets are in precise topographical ranges or whether a target is in a definite territory, is introduced in [11]. The approach presented by the authors is based on the coordinates of latitude and longitude produced by GNSS to discover the positions of these coordinates. The data records, including latitude and longitude in a popular social networking service platform, are employed in simulations, and the obtained results are encouraging.

The authors of [12] present an architecture for WSNs based on Sub1G-Hz and a star topology to reduce sensing nodes’ power consumption. The laboratory results determine that nodes’ packet error rate can adequately be commanded near a target value, proving beneficial communication reliability and keeping energy consumption low.

Finally, the guest editors are grateful to the Editor-in-Chief and the editorial staff of Sensors for accepting their special issue proposal and the kind cooperation, patience, and active engagement. 
